# Processing of DNA Ends in the Maintenance of Genome Stability

**DOI:** 10.3389/fgene.2018.00390

**Published:** 2018-09-12

**Authors:** Diego Bonetti, Chiara Vittoria Colombo, Michela Clerici, Maria Pia Longhese

**Affiliations:** Dipartimento di Biotecnologie e Bioscienze, Università di Milano-Bicocca, Milan, Italy

**Keywords:** checkpoint, DNA replication, double-strand break, MRX, nucleases, resection

## Abstract

DNA double-strand breaks (DSBs) are particularly hazardous lesions as their inappropriate repair can result in chromosome rearrangements, an important driving force of tumorigenesis. DSBs can be repaired by end joining mechanisms or by homologous recombination (HR). HR requires the action of several nucleases that preferentially remove the 5′-terminated strands at both DSB ends in a process called DNA end resection. The same nucleases are also involved in the processing of replication fork structures. Much of our understanding of these pathways has come from studies in the model organism *Saccharomyces cerevisiae*. Here, we review the current knowledge of the mechanism of resection at DNA DSBs and replication forks.

## Introduction

DNA double-strand breaks (DSBs) are highly cytotoxic forms of DNA damage because their incorrect repair or failure to repair causes chromosome loss and rearrangements that can lead to cell death or transformation (Liu et al., 2012). They can form accidentally during normal cell metabolism or after exposure of cells to ionizing radiations or chemotherapeutic drugs. In addition, DSBs are intermediates in programmed recombination events in eukaryotic cells. Indeed, defects in DSB signaling or repair are associated with developmental, immunological and neurological disorders, and tumorigenesis (O’Driscoll, 2012).

Conserved pathways extensively studied in recent years are devoted to repair DSBs in eukaryotes. The two predominant repair mechanisms are non-homologous end joining (NHEJ) and homologous recombination (HR) and the choice between them is regulated during the cell cycle. NHEJ allows a direct ligation of the DNA ends with very little or no complementary base pairing and it operates predominantly in the G1 phase of the cell cycle (Chiruvella et al., 2013). The initial step involves the binding to DNA ends of the Ku heterodimer, which protects the DNA ends from degradation, followed by ligation of the broken DNA ends by the DNA ligase IV (Dnl4/Lig4 in yeast) complex. By contrast, HR is the predominant repair pathway in the S and G2 phases of the cell cycle and it requires a homologous duplex DNA to direct the repair (Mehta and Haber, 2014). For HR to occur, the 5′-terminated DNA strands on either side of the DSB must first be degraded by a concerted action of nucleases to generate 3′-ended single-stranded DNA (ssDNA) tails in a process referred to as resection (Cejka, 2015; Symington, 2016). These tails are first bound by the ssDNA binding complex Replication Protein A (RPA). RPA is then replaced by the recombination protein Rad51 to form a right-handed helical filament that is used to search and invade the homologous duplex DNA (Mehta and Haber, 2014).

Double-strand break occurrence also triggers the activation of a sophisticated highly conserved pathway, called DNA damage checkpoint, which couples DSB repair with cell cycle progression (Gobbini et al., 2013; Villa et al., 2016). Apical checkpoint proteins include phosphatidylinositol 3-kinase related protein kinases, such as mammalian ATM (Ataxia-Telangiectasia-Mutated) and ATR (ATM- and Rad3-related), orthologs of *Saccharomyces cerevisiae* Tel1 and Mec1, respectively (Ciccia and Elledge, 2010). Once Mec1/ATR and/or Tel1/ATM are activated, their checkpoint signals are propagated through the *S. cerevisiae* protein kinases Rad53 and Chk1 (CHK2 and CHK1 in mammals, respectively), whose activation requires the conserved protein Rad9 (53BP1 in mammals) (Sweeney et al., 2005). While Tel1/ATM recognizes unprocessed or minimally processed DSBs, Mec1/ATR is recruited to and activated by RPA-coated ssDNA, which arises upon resection of the DSB ends (Zou and Elledge, 2003).

Most of our knowledge of the nucleolytic activities responsible for DSB resection has come from studies in the budding yeast *S. cerevisiae*, where DNA end resection can be monitored physically at sites of endonuclease-induced DSBs. Interestingly, the same nucleases involved in DSB resection are also responsible for the processing of stalled replication forks both in yeast and in mammals. Here we will focus on the work done in *S. cerevisiae* to understand the resection mechanism at DNA DSBs and replication forks and its regulation by Tel1/ATM and Mec1/ATR checkpoint kinases.

## Nuclease Action at Dna Double-Strand Breaks

Genetic studies in *S. cerevisiae* identified at least three distinct nucleases involved in end-resection: the MRX (Mre11-Rad50-Xrs2 in yeast; MRE11-RAD50-NBS1 in mammals) complex, Dna2 and Exo1 (DNA2 and EXO1 in mammals, respectively). In particular, the Mre11 subunit of MRX has five conserved phosphoesterase motifs in the amino-terminal half of the protein that are required for 3′–5′ double-strand DNA (dsDNA) exonuclease and ssDNA endonuclease activities of the protein *in vitro* (Bressan et al., 1998; Paull and Gellert, 1998; Trujillo et al., 1998; Usui et al., 1998). Rad50 is characterized by Walker A and B ATP binding cassettes located at the amino- and carboxy-terminal regions of the protein, with the intervening sequence forming a long antiparallel coiled-coil. The apex of the coiled-coil domain can interact with other MRX complexes by Zn^+^-mediated dimerization to tether the bound DNA ends together (de Jager et al., 2001; Hopfner et al., 2002; Wiltzius et al., 2005; Williams et al., 2008). The ATP-bound state of Rad50 inhibits the Mre11 nuclease activity by masking the active site of Mre11 from contacting DNA (Lim et al., 2011). ATP hydrolysis induces conformational changes of both Rad50 and Mre11 that allow the Mre11 nuclease domain to access the DSB ends and to be engaged in DSB resection (Lammens et al., 2011; Lim et al., 2011; Williams et al., 2011; Möckel et al., 2012; Deshpande et al., 2014).

**FIGURE 1:**
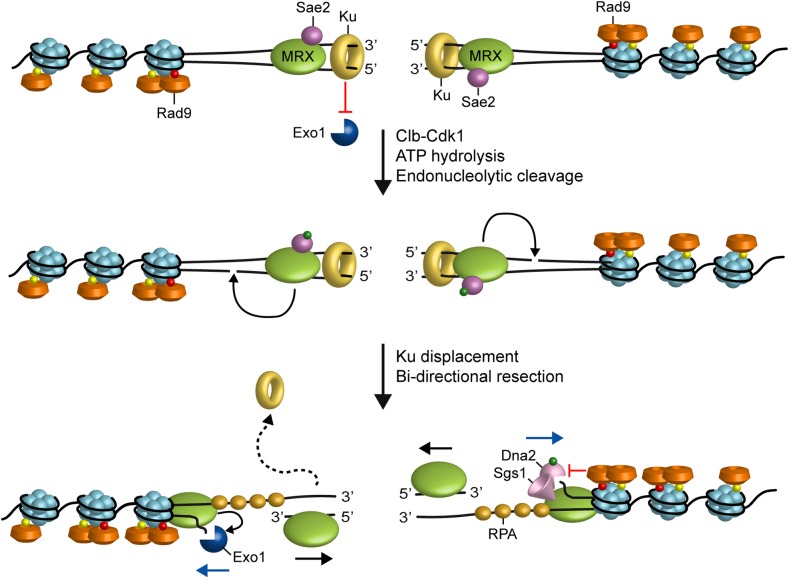
Model for resection of DNA DSBs. MRX, Sae2 and Ku are rapidly recruited to DNA ends. Ku inhibits Exo1 access to DNA ends. In the ATP-bound state, Rad50 blocks the Mre11 nuclease. After ATP hydrolysis by Rad50, Mre11 together with Sae2 phosphorylated by Cdk1 can catalyze an endonucleolytic cleavage of the 5’ strand. This incision allows processing by Exo1 and Sgs1-Dna2 in a 5’–3’ direction from the nick (blue arrows) and by MRX in a 3’–5’ direction toward the DSB ends (black arrows). MRX also promotes the association of Exo1 and Sgs1-Dna2 at DNA ends, whereas Rad9 inhibits the resection activity of Sgs1-Dna2. Red dots indicate phosphorylation events by Mec1 and Tel1, green dots indicate phosphorylation events by Cdk1 and yellow dots indicate methylation of histone H3.

In the current model for resection, the Sae2 protein (CtIP in mammals) activates a latent dsDNA-specific endonuclease activity of Mre11 within the context of the MRX complex to incise the 5′-terminated dsDNA strands at both DNA ends (Cannavo and Cejka, 2014). The resulting nick generates an entry site for the Mre11 exonuclease to degrade back to the DSB end in the 3′–5′ direction, and for Exo1 and Dna2 nucleases to degrade DNA in the 5′–3′ direction away from the DSB end (Mimitou and Symington, 2008; Zhu et al., 2008; Cejka et al., 2010; Niu et al., 2010; Garcia et al., 2011; Nimonkar et al., 2011; Shibata et al., 2014; Reginato et al., 2017; Wang et al., 2017; **Figure 1**). In yeast, inactivation of either Sgs1-Dna2 or Exo1 results in only minor resection defects, whereas resection is severely compromised when the two pathways are simultaneously inactivated, indicating that they play partially overlapping functions (Mimitou and Symington, 2008; Zhu et al., 2008).

The efficiency of 5′ DNA end cleavage *in vitro* by MRX-Sae2 was shown to be strongly enhanced by the presence of protein blocks at DNA ends (Cannavo and Cejka, 2014; Anand et al., 2016; Deshpande et al., 2016). It has been proposed that the endonucleolytic cleavage catalyzed by MRX-Sae2 allows the resection machinery to bypass end-binding factors that can be present at the break end and restrict the accessibility of DNA ends to Exo1 and Sgs1-Dna2. These end-binding factors includes Spo11, which cleaves DNA by a topoisomerase-like transesterase mechanism and remains covalently attached to the 5′ end of meiotic DSBs, trapped topoisomerases, or the Ku complex (see the next paragraph) (Neale et al., 2005; Bonetti et al., 2010; Mimitou and Symington, 2010; Langerak et al., 2011; Chanut et al., 2016).

While Exo1 shows 5′–3′ exonuclease activity capable to release mononucleotide products from a dsDNA end (Tran et al., 2002), Dna2 has an endonuclease activity that can cleave either 3′ or 5′ overhangs adjoining a duplex DNA (Kao et al., 2004). The resection activity of Dna2 relies on the RecQ helicase Sgs1 (BLM in humans) that provides the substrates for Dna2 by unwinding the dsDNA (Zhu et al., 2008; Cejka et al., 2010; Niu et al., 2010; Nimonkar et al., 2011). Furthermore, RPA directs the resection activity of Dna2 to the 5′ strand by binding and protecting the 3′ strand to Dna2 access (Cejka et al., 2010; Niu et al., 2010). In both yeast and humans, Dna2 contains also a helicase domain that can function as a ssDNA translocase to facilitate the degradation of 5′-terminated DNA by the nuclease activity of the enzyme (Levikova et al., 2017; Miller et al., 2017).

In addition to the end-clipping function, the MRX complex also stimulates resection by Exo1 and Sgs1-Dna2 both *in vitro* and *in vivo* (Cejka et al., 2010; Nicolette et al., 2010; Niu et al., 2010; Shim et al., 2010; Nimonkar et al., 2011). Biochemical experiments have shown that MRX enhances the ability of Sgs1 to unwind dsDNA, possibly by increasing Sgs1 association to DNA ends. Furthermore, MRX enhances both the affinity to DNA ends and the processivity of Exo1 (Cejka et al., 2010; Nicolette et al., 2010; Niu et al., 2010; Nimonkar et al., 2011; Cannavo et al., 2013). The MRX function in promoting Sgs1-Dna2 and Exo1 resection activities does not require Mre11 nuclease, suggesting that it does involve the Mre11 end-clipping activity (Shim et al., 2010).

Interestingly, MRX possesses an ATP-dependent unwinding activity capable of releasing a short oligonucleotide from dsDNA (Paull and Gellert, 1999; Cannon et al., 2013) and the recent identification of the hypermorphic *mre11-R10T* mutation has allowed us to demonstrate that this strand-separation function of MRX is important to stimulate Exo1 resection activity (Gobbini et al., 2018). In particular, Mre11-R10T mutant variant, whose single aminoacid substitution is located in the first Mre11 phosphodiesterase domain, accelerates DSB resection compared to wild type Mre11 by potentiating the processing activity of Exo1, whose association to DSBs is increased in *mre11-R10T* cells. Molecular dynamic simulations have shown that the two capping domains of wild type Mre11 dimer rapidly interact with the DNA ends and cause a partial unwinding of the dsDNA molecule. The Mre11-R10T dimer undergoes an abnormal rotation that leads one of the capping domain to wedge in between the two DNA strands and to persistently melt the dsDNA ends (Gobbini et al., 2018). These findings support a model in which MRX can directly stimulate Exo1 activity by promoting local unwinding of the DSB DNA end that facilitates Exo1 persistence on DNA. Although Exo1 is a processive nuclease *in vitro*, single-molecule fluorescence imaging has shown that it is rapidly stripped from DNA by RPA (Myler et al., 2016), suggesting that multiple cycles of Exo1 rebinding at the same DNA end would be required for extensive resection. Therefore, this MRX function in the stimulation of Exo1 activity at DNA ends can be of benefit to increase the processivity of Exo1 in the presence of RPA.

## Positive and Negative Regulation of Nuclease Action at Dna Double-Strand Breaks

Homologous recombination is generally restricted to the S and G2 phases of the cell cycle, when a sister chromatid is present as repair template (Aylon et al., 2004; Ira et al., 2004). This restriction is mainly caused by reduced end resection in G1 compared to G2. Reduced resection in G1 is due to both Ku binding to DNA ends and low cyclin-dependent kinase (Cdk1 in *S. cerevisiae*) activity (Aylon et al., 2004; Ira et al., 2004; Clerici et al., 2008; Zierhut and Diffley, 2008). Elimination of Ku in G1 (where Cdk1 activity is low) allows Cdk1-independent DSB resection that is limited to the break-proximal sequence, whereas the absence of Ku does not enhance DSB processing in G2 (where Cdk1 activity is high) (Clerici et al., 2008). Furthermore, inhibition of Cdk1 activity in G2 prevents DSB resection in wild type but not in *ku*Δ cells (Clerici et al., 2008). These findings suggest that Cdk1 activity is required for resection initiation when Ku is present. However, the finding that Cdk1 inhibition in G2-arrested *ku*Δ cells allows only short but not long-range resection (Clerici et al., 2008) suggests the existence of other Cdk1 targets to allow extensive resection. Consistent with this hypothesis, Cdk1 was shown to promote short- and long-range resection by phosphorylating and activating Sae2 and Dna2, respectively. In fact, substitution of Cdk1-dependent phosphorylation residues in Sae2 causes a delay of DSB resection initiation, while mutations of Cdk1-target sites in Dna2 cause a defect in long-range resection (Huertas et al., 2008; Huertas and Jackson, 2009; Manfrini et al., 2010; Chen et al., 2011).

Subsequent experiments have shown that the Ku complex is rapidly recruited to DSBs and protects the DNA ends from degradation by Exo1 (**Figure 1**). The absence of Ku partially suppresses both the hypersensitivity to DSB-inducing agents and the resection defect of *mre11*Δ and *sae2*Δ cells in an Exo1-dependent fashion (Mimitou and Symington, 2010; Foster et al., 2011; Langerak et al., 2011). This finding suggests that Sae2, once phosphorylated by Cdk1, promotes resection initiation by supporting MRX function in removing Ku from the DSB ends. As Ku preferentially binds dsDNA ends over ssDNA (Griffith et al., 1992), the MRX-Sae2 endonucleolytic activity could limit DSB association of Ku by creating a DNA substrate less suitable for Ku engagement (Mimitou and Symington, 2010; Langerak et al., 2011; Chanut et al., 2016). On the other hand, as the absence of MRX, but not of Sae2 or Mre11 nuclease activity, increases Ku association at DNA ends (Zhang et al., 2007; Wu et al., 2008; Shim et al., 2010), MRX could compete with Ku for end binding. However, the finding that hyperactivation of Exo1 resection activity by the Mre11-R10T mutant variant leads to Ku dissociation from DSB ends and Cdk1-independent DSB resection close to the DSB end suggests that MRX can limit Ku association to DNA ends also indirectly by promoting Exo1 resection activity (Gobbini et al., 2018).

In addition to Ku, the Rad9 protein, originally identified as adaptor for activation of Rad53 checkpoint kinase (Sweeney et al., 2005), inhibits DSB resection (Bonetti et al., 2015; Ferrari et al., 2015; **Figure 1**). The lack of Rad9 suppresses the resection defect of Sae2-deficient cells and increases the resection efficiency also in a wild type context (Bonetti et al., 2015; Ferrari et al., 2015). Both these effects occur in a Sgs1-Dna2-dependent fashion, indicating that Rad9 inhibits mainly the resection activity of Sgs1-Dna2 by limiting Sgs1 association to DSBs. Further support for a role of Rad9 in Sgs1-Dna2 inhibition comes from the identification of the hypermorphic Sgs1-G1298R mutant variant, which potentiates the Dna2 resection activity by escaping the inhibition that Rad9 exerts on Sgs1 (Bonetti et al., 2015).

Recruitment of Rad9 to chromatin involves multiple pathways. The TUDOR domain of Rad9 interacts with histone H3 methylated at K79 (H3-K79me) (Giannattasio et al., 2005; Wysocki et al., 2005; Grenon et al., 2007). Rad9 binding to the sites of damage is strengthened through an interaction of its tandem-BRCT domain with histone H2A phosphorylated at S129 (γH2A) by Mec1 and Tel1 checkpoint kinases (Downs et al., 2000; Shroff et al., 2004; Toh et al., 2006; Hammet et al., 2007). Finally, phosphorylation of Rad9 by Cdk1 leads to Rad9 interaction with the multi-BRCT domain protein Dpb11 (TopBP1 in mammals), which mediates histone-independent Rad9 association to the sites of damage (Granata et al., 2010; Pfander and Diffley, 2011).

Rad9 association to DSB ends is counteracted by the Swr1-like family remodeler Fun30 (SMARCAD1 in mammals) (Chen et al., 2012; Costelloe et al., 2012; Eapen et al., 2012; Bantele et al., 2017) and the scaffold protein complex Slx4-Rtt107 (Dibitetto et al., 2016; Liu et al., 2017), both of which promote DSB resection (Chen et al., 2012; Dibitetto et al., 2016). The Slx4-Rtt107 complex limits Rad9 binding near a DSB possibly by competing with Rad9 for interaction with Dpb11 and γH2A (Ohouo et al., 2013; Dibitetto et al., 2016).

## DNA Damage Checkpoint Regulation of Nuclease Action at Double-Strand Breaks

**FIGURE 2:**
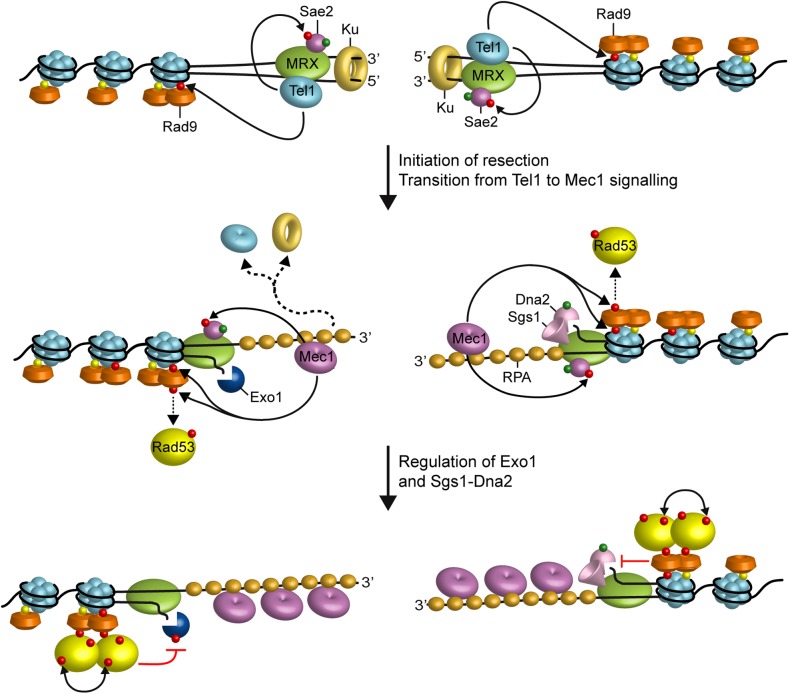
Interplays between end resection and checkpoint. Rad9 is already bound to chromatin via interaction with methylated histone H3 (yellow dots). When a DSB occurs, MRX, Sae2, and Ku localize to the DSB ends. MRX bound to DNA ends recruits and activates Tel1, which in turn promotes DSB resection by phosphorylating Sae2 and stabilizing MRX association to DNA ends. Tel1 also contributes to the recruitment of Rad9 to the DSB ends by phosphorylating H2A. Initiation of DSB resection by MRX-Sae2, Exo1, and Sgs1-Dna2 generate ssDNA tails that promotes a switch from Tel1 to Mec1 signaling. Activated Mec1 contributes to phosphorylate H2A that leads to a further enrichment of Rad9 at DSBs, which counteracts directly Sgs1-Dna2 resection activity. Mec1 also phosphorylates Rad9, which in turn allows Rad53 in-trans autophosphorylation and activation (double black arrows). Activated Rad53 limits DSB resection by phosphorylating and inhibiting Exo1. Phosphorylation events by Mec1 and Tel1 are indicated by red dots, whereas green dots indicate phosphorylation events by Cdk1.

Generation of DNA DSBs triggers the activation of the DNA damage checkpoint, whose key players include the *S. cerevisiae* protein kinases Mec1 (ATR in mammals) and Tel1 (ATM in mammals) (Gobbini et al., 2013). In both yeast and mammals, Tel1/ATM is activated by the MRX/MRN complex, which is required for Tel1/ATM recruitment to the site of damage through direct interaction between Tel1/ATM with the Xrs2 subunit (Nakada et al., 2003; Falck et al., 2005; Lee and Paull, 2005; You et al., 2005). By contrast, Mec1/ATR activation depends on its interactor Ddc2 (ATRIP in mammals) (Paciotti et al., 2000). While blunt or minimally processed DSB ends are preferential substrates for Tel1/ATM (Shiotani and Zou, 2009), RPA-coated ssDNA is the structure that enables Mec1/ATR to recognize DNA (Zou and Elledge, 2003). In both yeast and mammals, as the single-stranded 3′ overhangs increase in length, Mec1/ATR activation is coupled with loss of ATM/Tel1 activation, suggesting that DSB resection promotes a switch from a Tel1/ATM- to a Mec1/ATR-dependent checkpoint (Mantiero et al., 2007; Shiotani and Zou, 2009; **Figure 2**). The substrates for Mec1 and Tel1 are largely overlapping and include H2A, Rad53/CHK2, Chk1, Rad9/53BP1, Sae2/CtIP, Dna2, and RPA (Ciccia and Elledge, 2010).

The DNA damage checkpoint regulates the generation of 3′–ended ssDNA at DNA ends in both positive and negative fashions. Cells lacking Tel1 slightly reduce the efficiency of DSB resection (Mantiero et al., 2007). Tel1, which is loaded at DSBs by MRX, supports MRX persistence at DSBs in a positive feedback loop (Cassani et al., 2016), suggesting that it can facilitates DSB resection by promoting MRX function. Interestingly, Tel1 exerts this role independently of its kinase activity (Cassani et al., 2016), suggesting that it plays a structural role in stabilizing MRX retention to DSBs.

In contrast to *tel1*Δ cells, cells lacking Mec1 accelerate the generation of ssDNA at the DSBs, whereas the same process is impaired by the *mec1-ad* allele (Clerici et al., 2014), indicating that Mec1 inhibits DSB resection. Mec1 exerts this function at least in two ways: (i) it induces Rad53-dependent phosphorylation of Exo1 that leads to the inhibition of Exo1 activity (Jia et al., 2004; Morin et al., 2008), (ii) it promotes retention of the resection inhibitor Rad9 at DNA DSBs through phosphorylation of H2A on serine 129 (Eapen et al., 2012; Clerici et al., 2014; Gobbini et al., 2015). The association of Rad9 at DSBs and therefore the inhibition of DSB resection is promoted also by the checkpoint sliding clamp Ddc1-Mec3-Rad17 (9-1-1 in mammals) complex (Ngo and Lydall, 2015), which is required for full Mec1 activation and binds to the ssDNA-dsDNA junction at DNA ends (Gobbini et al., 2013).

Both Mec1 and 9-1-1 have also a positive role in DSB resection. In fact, Mec1 is known to phosphorylate Sae2 and this phosphorylation is important for Sae2 function in resection of both mitotic and meiotic DSBs (Baroni et al., 2004; Cartagena-Lirola et al., 2006). Furthermore, Mec1 also phosphorylates Slx4 and this phosphorylation favors DSB resection by promoting Dpb11-Slx4-Rtt107 complex formation that leads to a destabilization of Rad9 association at DSBs (Smolka et al., 2007; Ohouo et al., 2013; Dibitetto et al., 2016).

Finally, in the absence of Rad9, the 9-1-1 complex facilitates DSB resection by stimulating both Dna2-Sgs1 and Exo1 through an unknown mechanism (Ngo et al., 2014). This effect of 9-1-1 is conserved, as also the human 9-1-1 complex stimulates the activities of DNA2 and EXO1 *in vitro* (Ngo et al., 2014).

In yeast, the checkpoint response to DNA DSBs depends primarily on Mec1. However, if resection initiation is delayed, for example, in the *sae2*Δ mutant, MRX persistence at DSBs is increased, Tel1 is hyperactivated and the *mec1*Δ checkpoint defect is partially bypassed (Usui et al., 2001; Clerici et al., 2006). This persistent checkpoint activation caused by enhanced MRX and Tel1 signaling activity at DSBs contributes to the DNA damage hypersensitivity and the resection defect of Sae2-deficient cells by increasing Rad9 persistence at DSBs. In fact, *mre11* mutant alleles that reduce MRX binding to DSBs restore DNA damage resistance and resection in *sae2*Δ cells (Chen et al., 2015; Puddu et al., 2015; Cassani et al., 2018). Furthermore, reduction in Tel1 binding to DNA ends or abrogation of its kinase activity restores DNA damage resistance in *sae2*Δ cells (Gobbini et al., 2015). Similarly, impairment of Rad53 activity either by affecting its interaction with Rad9 or by abolishing its kinase activity suppresses the sensitivity to DNA damaging agents and the resection defect of *sae2*Δ cells (Gobbini et al., 2015). The bypass of Sae2 function by Rad53 and Tel1 impairment is due to decreased amount of Rad9 bound at the DSBs (Gobbini et al., 2015). As Rad9 inhibits Sgs1-Dna2 (Bonetti et al., 2015; Ferrari et al., 2015), reduced Rad9 association at DSBs increases the resection efficiency by relieving Sgs1-Dna2 inhibition.

Altogether, these findings support a model whereby the binding of MRX to DNA ends drives the recruitment of Tel1, which facilitates initiation of end resection by phosphorylating Sae2 and promoting MRX association to DNA ends (**Figure 2**). Generation of RPA-coated ssDNA leads to the recruitment of Mec1-Ddc2, which in turn phosphorylates Rad9, Rad53, and H2A. γH2A generation promotes the enrichment of Rad9 to the DSB ends, which limits the resection activity of Dna2-Sgs1. Rad9 association at DSBs also leads to the inhibition of Exo1 activity indirectly by allowing activation of Rad53, which in turn phosphorylates and inhibits Exo1 (**Figure 2**).

This Mec1-mediated inhibition of nuclease action at DSBs avoids excessive generation of ssDNA, which can form secondary structures that can be attacked by structure-selective endonucleases, leading to chromosome fragmentation. Furthermore, since Mec1 is activated by RPA-coated ssDNA, inhibition of end resection by Mec1 keeps under control Mec1 itself. This negative feedback loop may avoid excessive checkpoint activation to ensure a rapid checkpoint turning off to either resume cell cycle progression when the DSB is repaired or adapt to DSBs as a final attempt at survival after cells have exhausted repair options.

## Nuclease Action at the Replication Forks

Accurate and complete DNA replication is essential for the maintenance of genome stability. However, progression of replication forks is constantly challenged by various types of replication stress that generally causes a slowing or stalling of replication forks (Giannattasio and Branzei, 2017; Pasero and Vindigni, 2017). Replication forks can slow or stall at sites containing DNA lesions, chromatin compaction, DNA secondary structures (G-quadruplex, small inverted repeats, trinucleotides repeats), DNA/RNA hybrids and covalent protein-DNA adducts. Furthermore, clashes between transcription and replication machineries can impact genome stability even in unchallenged conditions (Giannattasio and Branzei, 2017; Pasero and Vindigni, 2017). Fork obstacles may result in dysfunctional replication forks, which lack their replication-competent state and necessitate additional mechanisms to resume DNA synthesis. Failure to resume DNA synthesis results in the generation of DNA DSBs, a major source of the genome rearrangements (Liu et al., 2012).

A general feature of stalled replication forks is the accumulation of ssDNA that can originate from physical uncoupling between the polymerase and the replicative helicase or between the leading and the lagging strand polymerases (Pagès and Fuchs, 2003; Byun et al., 2005; Lopes et al., 2006). The accumulation of torsional stress ahead of replication forks (Katou et al., 2003; Bermejo et al., 2011; Gan et al., 2017) can also lead to the annealing of the two newly synthesized strands and the formation of a four-way structure resembling a Holliday junction (i.e., fork reversal), which might expose DNA ends to exonucleolytic processing (Sogo et al., 2002). These tracts of ssDNA coated by the RPA complex recruit the checkpoint kinase Mec1/ATR (Zou and Elledge, 2003), whose activation prevents entry into mitosis, increases the intracellular dNTP pools, represses late origin firing, maintains replisome stability and orchestrates different pathways of replication fork restart/stabilization (Giannattasio and Branzei, 2017; Pasero and Vindigni, 2017).

In both yeast and mammals, the same nucleases involved in DSB resection are emerging as key factors for the processing of replication intermediates to allow repair/restart of stalled replication forks and/or to prevent accumulation of DSBs (Cotta-Ramusino et al., 2005; Segurado and Diffley, 2008; Tsang et al., 2014; Thangavel et al., 2015; Colosio et al., 2016). Indeed, the ability of Mre11, Sae2, Dna2, and Exo1 to resect dsDNA ends is relevant to prevent the accumulation of replication-associated DSBs by promoting DSB repair by HR (Costanzo et al., 2001; Cotta-Ramusino et al., 2005; Segurado and Diffley, 2008; Hashimoto et al., 2012; Tsang et al., 2014; Yeo et al., 2014; Thangavel et al., 2015; Colosio et al., 2016; Ait Saada et al., 2017). In addition, controlled Dna2-mediated degradation of replication forks is a relevant mechanism to mediate reversed fork restart (Thangavel et al., 2015).

Although the nucleolytic processing of nascent strands at stalled replication forks is important to resume DNA synthesis, unrestricted nuclease access can also promote extensive and uncontrolled degradation of stalled replication intermediates and genome instability (Pasero and Vindigni, 2017). In budding yeast, the checkpoint activated by the ssDNA that arise at stalled replication forks plays a role in protecting replication intermediates from aberrant nuclease activity (Tercero and Diffley, 2001; Alabert et al., 2009; Barlow and Rothstein, 2009). In fact, in the absence of the checkpoint, relieve of Exo1 inhibition by Rad53 leads to the formation of long ssDNA gaps and fork collapse (Sogo et al., 2002; Cotta-Ramusino et al., 2005; Segurado and Diffley, 2008). Furthermore, replication stress in ATR-defective *Schizosaccharomyces pombe* and mammalian cells results in MRE11- and EXO1-dependent ssDNA accumulation (Hu et al., 2012; Koundrioukoff et al., 2013; Tsang et al., 2014). Interestingly, in *S. cerevisiae*, Tel1/ATM was recently found to counteract nucleolytic degradation by Mre11 of replication forks that reverse upon treatment with camptothecin (CPT) (Menin et al., 2018), which leads to accumulation of torsional stress by blocking Top1 on DNA (Postow et al., 2001; Koster et al., 2007; Ray Chaudhuri et al., 2012). Fork reversal in CPT is promoted by the replisome component Mrc1, whose inactivation prevents fork reversal in both wild type and *TEL1* deleted cells (Menin et al., 2018).

Interestingly, the same negative regulators of DSB resection limit nuclease action also at the replication forks. In yeast, Rad9, which is known to counteract the resection activity of Sgs1-Dna2, is important to protect stalled replication forks from detrimental Dna2-mediated degradation when Mec1/ATR is not fully functional (Villa et al., 2018). This Rad9 protective function relies mainly on the interaction of Rad9 with Dpb11, which is recruited to stalled replication forks at origin-proximal regions (Balint et al., 2015). Similarly, human cells lacking 53BP1, the mammalian Rad9 ortholog, are hypersensitive to DNA replication stress and show degradation of nascent replicated DNA (Her et al., 2018). Furthermore, the Ku heterodimer was shown to be recruited to terminally arrested replication forks and to regulate their resection in *S. pombe* (Teixeira-Silva et al., 2017). The lack of Ku leads to extensive Exo1-mediated fork resection, a reduced recruitment of RPA and Rad51 and a delay of fork-restart, suggesting that arrested replication forks undergo fork reversal that provides a substrate for Ku binding.

In addition to the checkpoint, other proteins are devoted to protect replication forks from degradation in mammalian cells. The absence of proteins involved in HR or in the Fanconi anemia network, including FAN1, FANCD2, RAD51, BRCA1, and BRCA2, leads to uncontrolled DNA degradation by MRE11 and EXO1 (Howlett et al., 2005; Hashimoto et al., 2010; Schlacher et al., 2011, 2012; Ying et al., 2012; Chaudhury et al., 2014; Karanja et al., 2014; Chen et al., 2016; Kais et al., 2016; Kolinjivadi et al., 2017; Lemaçon et al., 2017; Mijic et al., 2017; Taglialatela et al., 2017). Furthermore, loss of the WRN exonuclease activity enhances degradation at nascent DNA strands by EXO1 and MRE11 (Su et al., 2014; Iannascoli et al., 2015), whereas cells depleted of the biorientation defect 1-like (BOD1L) protein exhibit a DNA2-dependent degradation of stalled/damaged replication forks (Higgs et al., 2015).

## Conclusion

Defects in HR and DNA replication underlie a significant proportion of the genomic instability observed in cancer cells. Furthermore, ssDNA formed at DSBs and at replication forks can be source of clustered mutations, frequently occurring during carcinogenesis, and of error-prone repair events that can cause DNA deletions or translocations (Nik-Zainal et al., 2012; Roberts et al., 2012; Alexandrov et al., 2013; Sakofsky et al., 2014). Therefore, there is a growing interest in understanding how ssDNA is generated at both DSBs and replication forks and how its generation is regulated. Mounting evidence indicates that processing of both DSB ends and replication forks is regulated both positively and negatively by several proteins involved also in the DNA damage checkpoint, thus coupling resection with checkpoint activation. Given the importance to maintain genome stability, advancements in delineating the mechanisms that control nuclease action at both DSBs and replication forks will have far-reaching implications for human health.

## Author Contributions

MPL conceived the idea. DB and MPL wrote the manuscript. CVC and MC revised and edited the manuscript.

## Conflict of Interest Statement

The authors declare that the research was conducted in the absence of any commercial or financial relationships that could be construed as a potential conflict of interest.
